# Clinical Holistic Medicine: Whiplash, Fibromyalgia, and Chronic Fatigue

**DOI:** 10.1100/tsw.2005.46

**Published:** 2005-04-25

**Authors:** Søren Ventegodt, Mark Gringols, Joav Merrick

**Affiliations:** ^1^ The Quality of Life Research Center, Teglgårdstræde 4-8, DK-1452 Copenhagen K, Denmark; ^2^ Clalit Health Services and National Institute of Child Health and Human Development, Ben Gurion University, Beer-Sheva, Israel; ^3^ National Institute of Child Health and Human Development, Office of the Medical Director, Division for Mental Retardation, Ministry of Social Affairs, Jerusalem and Zusman Child Development Center, Division of Pediatrics and Community Health, Ben Gurion University, Beer-Sheva, Israel

**Keywords:** quality of life, QOL, philosophy, human development, holistic medicine, public health, holistic health, holistic process theory, life mission theory, group therapy, Denmark

## Abstract

Holistic treatment of the highly complex, “new diseases” are often possible with the tools of consciousness-based medicine. The treatment is more complicated and the cure usually takes longer than for less-complex diseases. The problem with these patients is that they have less easily accessible resources than most patients, as they suffer from a combined socio-psycho-physical problem with depression, poor social standing, low confidence, and low self-esteem. Often, they have also already tried most of the specialist and alternative treatments on the market. To cure them, the most important thing is to coach them to improve their social life by changing their behavior to be of more value to others. Holding and processing must be especially careful and the contract with the patients must be extremely explicit in order to work on their personal development for 6—12 months. The new diseases can be cured with consciousness-based medicine if the patients are motivated and keep their appointments and agreements. Low responsibility, low personal energy, little joy of life, and limited insight into self and existence are some of the features of the new diseases that make them difficult to cure. The important thing is to keep a pace the patient can follow and give the patient a row of small successes and as few failures as possible. The new diseases are a challenge, a unique chance to improve communication, holding, and processing skills.

## INTRODUCTION

In recent years, a number of "new diseases" has emerged. These are suspected of being lifestyle diseases that are particularly characteristic of our western culture. The best known are whiplash, fibromyalgia, and chronic fatigue syndrome. These diseases appear to be nonexistent in many indigenous cultures, such as those in Africa, and the cause of these diseases is a mystery. The “new diseases” are characteristically vague, which makes them technically difficult to diagnose. In addition, they seem to be related to some intense, peculiar, and inscrutable personality disorders that can make the patient look like one major apology for existing or even a social outcast who is just looking for a good alibi to avoid all social and human commitments. Accordingly, many physicians do not recognize the new diseases as being real diseases and understand them as mere social problems or personality disorders.

From a holistic perspective, illness can be any conglomerate of poor posture, emotional pain, and physical blockages, so we see no difficulty in acknowledging the new diseases as a form of illness. The holistic medicine explanation allows the new diseases to be treated and the treatment is often surprisingly effective. Before we discuss the three illnesses and their suggested holistic cures, we will give a short review of our previous work in the field of consciousness-based medicine — or “quality of life as medicine” — as we often call our style of holistic medicine. With the new diseases many new and alternative treatments have come into use; unfortunately we have recently found therapies like gestalt therapy, Marion Rosen therapy, and Cranio Sacral therapy to be of little value to chronic WAD patients[1]. In this paper we present the method of holistic existential therapy, which efficiency still needs to be documented.

## CLINICAL HOLISTIC MEDICINE

The life mission theory[2,3,4,5,6] states that everybody has a purpose of life or huge talent. Happiness comes from living this purpose and succeeding in expressing the core talent in life. To do this, it is important to develop into what is known as the natural condition, a condition where the person knows himself and uses all his efforts to achieve what is most important for him. The holistic process theory of healing[7,8,9,10] and the related quality of life theories[11,12,13] state that the return to the natural state of being is possible whenever the person gets the resources needed for existential healing. The resources needed are “holding” in the dimensions of awareness, respect, care, acknowledgment, and acceptance with support and processing in the dimensions of feeling, understanding, and letting go of negative attitudes and beliefs. The preconditions for the holistic healing to take place are trust and the intention for the healing to take place. Existential healing is not a local healing of any tissue, but a healing of the wholeness of the person, making him much more resourceful, loving, and knowledgeable of himself, his own needs, and his wishes. In letting go of negative attitudes and beliefs, the person returns to a more responsible existential position and an improved quality of life. The philosophical change of the person healing is often a change towards preferring difficult problems and challenges instead of avoiding difficulties in life[14,15,16,17,18,19,20,21]. The person who becomes happier and more resourceful often also becomes more healthy, more talented, and more able to function[22,23,24].

## WHIPLASH: STATUS OVER A TYPICAL “NEW DISEASE”

For some years, there has been an intense debate on the nature of chronic whiplash-associated disorders (WAD)[25]. The Quebec Task Force Classification defined five groups for the acute phase of WAD[26] with most of the whiplash patients healing spontaneously, but some continuing into a chronic phase, reported by different researchers from 0% to above 30%[26,27,28,29].

In the acute phase, immediately after the physical impact and the whip-like movement of the spine, everybody seems to agree that there might be some physical damage to the muscles, bones, joints, tendons, fascia, and the connective tissue, even in the case of no obvious fracture or other gross damage. The issue of discussion is why these often small and dispersed damages are followed by symptoms that last for years. One theory is that high quality of life is associated with less worry about the physical pain, resulting in better healing[30]. One study demonstrated a number of minor damages, like small cracks in the bones[31], but most MRI (magnetic resonance imaging) studies have not shown any sign of significant damage[32] in the initial phase, except for one study that found minor whiplash-associated damages to the disc in a small percentage of the patients[33].

The chronic phase has been difficult to understand. One group worked with the theory that the facet joints were causing the pain and found that about half of the patients had a positive effect from local anesthesia[34,35]. Other groups found small diversities from normal in rotation pattern[36] or eye movements[37,38], but it was very difficult to establish a single, well-defined organic damage to blame for the diversity of WAD symptoms found. Our group of patients[25] had the typical cocktail of symptoms: pains; difficulties in moving the head, neck, and back; vertigo; low personal energy; lack of concentration; lack of motivation; sleeping disturbances; lack of sexual interest and energy; and a general feeling of being sick.

It is believed that psychological factors are of major importance in WAD. One study showed that the most important predictor for pain and discomfort at a later stage was the patient’s insurance situation, because if the patient was going to fight for insurance money, it was very likely that he or she would have WAD symptoms at a later stage[39]. In countries with less insurance, the average healing time was much shorter[26,40,41,42]. There has been critique of the conclusions because the number of participants was small[43] and because of difficulties in comparing the technical circumstances of the incidents[44]. Another indicator of the importance of psychological factors in WAD was the fact that the threshold for sensing pain is dramatically increased with growing mental involvement[27,45]. We all know the extreme situations, like when you were relaxing at the beach and an ant bite felt like a lion bite or, on the other extreme, the soldier at war continuing to fight in spite of being wounded. The memory of an old wound, now healed, can even cause pain[46,47]. The whole area of psychosomatics is well described in medical science, as are many psychoimmunological factors. A poststress syndrome is found to be a prognostic factor[48]. From the present knowledge, we are forced to conclude that the etiology of WAD is still not well understood and an important contribution from psychological and existential factors must be expected.

In the acute phase, there are several methods in use from immobilization[49], “live as usual” programs[50], mobilizations[51], to exercise[52], but it is not clear if any of these has any preventative effect for the later development of a chronic phase of WAD. The best advice in the acute phase at the moment seems to be to live as usual, as exercise is better than the cervical collar[53] still used for 85% of the patients in, for example, Germany[54]. For the chronic phase of WAD there is no cure available.

## FIBROMYALGIA

This syndrome of unknown etiology is characterized by chronic extensive pain, increased tenderness to palpation, and additional symptoms such as disturbed sleep, stiffness, fatigue, and psychological distress[55,56]. In spite of a reasonable amount of research, little progress has been made in the treatment during the last decade and very few trials have actually assessed improvement in functional status[57], as the relief of the chronic pain has been the major ambition of most interventions.

The complexity of the syndrome causes many researchers to recommend a multidisciplinary treatment program[55], but unfortunately, there is no evidence that a multimodal mind-body intervention program is better than education and support[58]. Many different approaches have been tried, often with modest effect on only about a third of the patients, like exercise[59], ultrasound[58], pool exercise therapy[60], and biofeedback[61]. Biomedicine has shown very little effect; amitriptyline and low-power laser therapy gave clinically significant results, but only about 30% of the patients responded[62,63,64,65, 66]. Serotonin reuptake inhibitors, NSAIDs, and corticosteroids have not been effective[64].

A psychological approach has shown similarly poor results, but making the patients aware of their basic psychological problems might be helpful to some patients[61], whereas integrated group therapy might be helpful to others[65].

Patients with fibromyalgia have painful tensions and blockages throughout their bodies. Conventional medicine works with a number of hypotheses for the disease, of which the autoimmune hypothesis (the hypothesis of myositis) is probably the most interesting. Psychologically, these patients often insist on the strategy of attributing all complaints, including emotional ones, to physical causes. One possible explanation of this tendency is that their emotional pain can be much worse to experience and relate to than their physical pain. From a holistic medical perspective, a patient with fibromyalgia does not become well until he, or more often *she*, is willing to feel the emotional pain, relate to it, and act on it[9]. If the patient is willing to assume responsibility for the pain, treatment is possible and through a persistent and targeted effort, the patient can regain mobility and normal capacity for work in a matter of years.

These patients can become free from pain, but this necessitates a comprehensive healing process where they work on the total pain load that they have accumulated during their personal lives. In short, it appears that these patients can become well again under professional holistic guidance if they are willing to fight for it. As with many holistic therapies, there is insufficient scientific evidence of the effect of this therapy. The Quality of Life Research Centre is currently carrying out a number of projects to gain evidence and is trying to obtain funding for systematic studies of the effect of holistic medicine on the numerous diseases where it apparently has an effect.

> **Male, aged 45 years, with fibromyalgia:** First visit: 1. Presents with tightness in the chest and diffuse symptoms of low well-being. He has been coughing for 21 days and running a temperature nine days ago. Stopped smoking four years ago. Apparently he has a history of fibromyalgia and collapse of vertebrae and a tendency towards depression. Previously treated twice with antidepressants. In addition, he takes antidepressants, anxiolytics and receives physiotherapy. On examination: Normal mood. Auscultation of the lungs: everywhere vesicular respiration with no abnormal sounds, slightly “asthmatic” respiration, peak flow 360, 370, 390 (normal range 410–540). Cardiac auscultation: Irregular pulse, suspected arrhythmia? BP: 120/90. Apparently no fever. Trigger-point test and medical history point to /fibromyalgia/. The acute deterioration is probably due to /suspected influenza/. 2. The patient worries that his depression may return; we shall help him with that. Can return for conversation. 3. He has been out of the labour market for 16 years. Now his children are grown up and he wants to get back and train for work.
>
> Second visit: Conversation: He is showing good progress. He is “pumping iron” at the physiotherapy clinic, but not finding it easy. He talks about his insectophobia, and I give him the EXERCISE to sit for 5–10 minutes and feel his fear, instead of running about screaming as he is doing at present. He can do this with other feelings that suddenly overwhelm and upset him. The important thing is for him to be in control, and to learn in a constructive manner to accommodate his feelings. I prepare him for working on his depression in the same way, should it appear again. On examination: rather apathetic. Will postpone training for work until we have found a way to do it.
>
> Third visit: His entire body is sore from mowing the grass two days ago. Massaging of tender muscles. We talk about the physiotherapy: he is still progressing and likes doing it, but feels guilty at spending all that money. He also swims, which costs money too. He does not like to spend too much. I ask whether his wife is finding that a problem, which is far from the case. Then I ask him what he believes his wife would prefer: that he becomes well, or that he saves the money. He becomes cross with me: “Don’t make me feel guilty. I’ve felt guilty all my life.” We talk about his feeling of guilt and how he can move beyond it. It is important that he allows himself the treatment he needs. He makes another appointment.

As mentioned, patients with fibromyalgia can be rather sad people with a mixture of serious, sometimes disabling, physical, mental, and social problems that are characteristic of the disease, combined with a rare ability to do nothing about them. Difficulties accumulate because of the patient's failure to assume responsibility for his or her own life and lack of self-discipline. The aim of the regime is to treat the apathy or defeatist attitude hidden in the mentality of these patients and this can prove difficult if the patient is unwilling to look below the surface, assume responsibility, and work with him- or herself. Once the patient has decided to become well and is willing to pay the price in the form of working intensively, fibromyalgia becomes "simple to treat". However, the treatment can be a protracted affair, taking 6 months, 12 months, or even a decade with the focus on personal development following the holistic regime.

> **Female, aged 51 years, with fibromyalgia, severe overweight, depression, and social phobia:** First quality-of-life (QOL) conversation: 1. Fibromyalgia: The patient suffers greatly from pain in her legs, thighs and arms and wants to be free from pain. 2. Severe overweight; today she weighs 138 kg (dressed). Normal weight 65 kg; since 1985 when she gave birth her weight has gone up. 3. Depression, for which she takes Cipramil [citalopram] 60 mg daily plus a sedative 20 mg in the evening – she has had death wish – and she also had a history of social phobia. We talk about how life hurts, and how we can only obtain physical freedom from pain by being willing to feel the pain emotionally. We have a long and deep conversation about this, and the patient cries a little, while reflecting on this. She does not want to go on living, she says, but in reality this is not about a death wish, but merely a resignation with regard to living. She is constantly gaining weight and no longer weighs herself. We have a good talk about life philosophy and agree that the patient should have 6–10 sessions of Rosen therapy, after which she can return to me (SV). We believe that all four major problems have the same underlying cause, which gives us hope that we can deal with all four diseases in one go, if the patient acknowledges the unavoidable pain of life and balances it with enjoyment of life. We talk about enjoyment of life lying at the very bottom beneath all the blockages, and the patient seems to understand this.
>
> Second QOL conversation: We talk about "how to eat an elephant" – the patient's insurmountable problems which keep growing – which you do one bite at a time. The elephant is the patient's neurosis, which feeds on her life energy and grows, because she keeps feeding it. That is how she loses energy. We talk about how big the elephant is at present and how small she is, and the patient says that it is 4 metres, while she herself is only half a metre. Six-month objective: The patient should become at least as big as the elephant so that it can no longer be in charge of her life. We agree that she should end up being slightly bigger than the elephant, and she should know how to catch and eat an elephant by then so that her positive development will continue after she finish in our clinic. If she succeeds, she will get her life back and have set a course to continue improving it. Basic work includes perception, conduct of life and the reestablishment of the patient's self-respect. She promises to do her exercises and follow the treatment for six months. I (SV) promise that we will give her all the help and support she needs during this period. The patient understands that she herself has to do the work involved in recovering; she is responsible for getting back on her feet – we provide help for self-help. She (her white side) allies herself with us to deal with the problems that she would be powerless to confront on her own. Agreed: The patient is to work with herself two hours daily. EXERCISE: Life story – write down all emotional events; what happened, how did you feel, what did you think, did you make any decisions. Start from the present.

> Third QOL conversation: Since last session the patient has begun to work out. Her fibromyalgia pain is no better, but is less significant and controls her less. She has begun to lose weight and today weighs 135 kg. She has done all her homework well. Her objective, which we correct below, and personal history are full of disappointments. We talk about confidence: it appears as though the pattern has been broken. So if she could let go of her basic lack of confidence in herself, other people and life itself, she would see life in its proper perspective. She makes the following statement: "They don't want what's good" – other people cannot be trusted. "You cannot trust anyone", "Everything looks hopeless", "I am hopeless", "I am stupid", "I am disappointed". The patient lacks self-respect. On the shopping list: new backbone. Weekly weighing here in clinic. EXERCISE: Write your life story including above statements. A topic for each day: write down all events, afterwards you can let go of the statement. She wants to talk more before Rosen treatment[1]. Another appointment is made.
>
> Fourth QOL conversation: She is showing progress in spite of everything. Talks about the Rosen sessions. She can talk with the Rosen practitioner to put her mind at ease. Weight today: 134.0 kg. Dietary advice: Eat vegetables, fruit in moderation and lean meat. Vegetables: eat as much as you like of cucumber, tomatoes, lettuce, asparagus, celery, fennel and cabbage. Avoid: fatty foods (ice-cream), potatoes, rice and bread, sweets and chocolate. The patient is getting more exercise – gym work, swimming – try a center, which has hot-water baths. Takes a 30–60-min walk every evening. Sauna against pain. Concerning social phobia – talks only to an elderly man on her stairway who calls her and her children. EXERCISE: Always trying to avoid contact. Now do the opposite: make yourself available, go and sit in the park and talk to people who come up to you. PLAN: Rosen method six times a week, then clinic again, perhaps followed by gestalt.
>
> Fifth QOL conversation: She had been to Rosen therapy three times, which has gone really well. She says: "it is strange to be understood; I'm not used to that". Now she has reached a point where it does not feel so good, but makes her want to run away. "It's as if I'd rather be invisible." We talk about that – when her feelings are strong, she is scared of being condemned. We talk about offender, victim, helper/deserter and self-torturer. Remembers hitting her doll. Acknowledges that she is a teeny bit evil. She understands that - "it's scarily human," she says. She is aware that she is protecting herself by becoming invisible, but then is not herself, which is very bad for her. She is pleased with the result so far.
>
> Ninth QOL conversation: Weight today: 114 kg. She has lost 22 kg of the 25 kg she had set as her target. "Wow, am I good," she says grudgingly, laughing. Social phobia – she can talk to people now, but still has periods when she keeps to herself. Right now she has come out of her isolation. Fibromyalgia – a great deal of pain in thighs and legs, arms and back. Her suppression of herself and all pleasure is what causes the muscle pain. Depression – her psychiatrist has prescribed 60 mg citalopram daily. Currently no symptoms, but when the medication is tapered she has thoughts of not wanting to live. Once she is able to enjoy things more she should gradually reduce dosage to 30 mg together with psychiatrist and then stop taking medication. All in all she is showing good progress.

After 9 months, this patient has almost reached her target: to become bigger and more powerful than her neurosis, symbolized as an internal elephant at the beginning of therapy. Now it is only slightly bigger than her and her favorable development is continuing. It appears as though she is getting there.

## CHRONIC FATIGUE SYNDROME

Generally, biomedicine has not been of much help to patients with chronic fatigue syndrome (CFS). Intravenous immunoglobulin was used 20 years ago, but it was not very effective, as only 31% (over placebo) of the patients were somewhat helped[67]. Hydrocortisone and fludrocortisone[68], melatonin or phytotherapi[69], acyclovir [70], tricyclic antidepressants, and other pharmacological treatments have generally not been effective[71]. Cognitive therapy (CT) has been a little helpful, guided support groups less efficient, but the best treatment so far seems to be CT even though only 27% of the patients reported improvements in one study[72]. Cognitive behavior therapy is definitely not a cure, as only every third of the patients benefit clinically in the most favorable studies[73,74,75]. A review concluded that "there is no satisfactory evidence for the effectiveness of CT in patients with the milder forms of CFS found in primary care or in patients who are so disabled that they are unable to attend out-patients"[76]. For teenagers 11–18 years of age, family therapy seemed helpful[77]. It is obvious that CFS is a very difficult syndrome to treat with an uncertain etiology, but several theories exist from immunological[69] to existential.

One important concept in holistic medicine is "somatization" (literally meaning "making bodily"). Somatization comes from our freedom to choose our own content of our consciousness and, thus, our freedom to repress unwanted feelings, thoughts, perceptions, and other existential material. In this repression, our mental, existential, and emotional problems take on a physical manifestation. Often, we find it easier to bear our problems as a physical ailment rather than as an emotional or existential problem, as we see with many patients in the holistic clinic. It is, therefore, logical to regard CFS as a somatized depression. Depression is the result of handling a major collection of repressed emotional pain systematically by taking the life-limiting decision (or generalized justification) that one is worth nothing, that one has very little self-confidence, and can cope with very little. By systematically invalidating our own self-esteem and self-confidence, we become dull, slow, sad, and emotionally dead — in other words, depressed. Depression is painful in itself, so we can flee from that too, which we do by banishing the depression to the body. In this way, virtually all feelings and all life energy have been banished to an area beyond our consciousness and the result is a total loss of personal energy: CFS. Below, two case stories are presented to illustrate the condition.

> **Male, aged 25 years, with lethargy:** First QOL conversation: The patient is suffering from serious problems with pain and tension in shoulders, "oxygen deficit"-like lethargy and physical weakness (including an urge to sleeping). On examination: a palpable muscle knot of 1.5 x 1.5 cm corresponding to C3-C4 (right side). Other problems: General scepticism about people's ulterior motives – seems not to read their intentions. Some shyness is easily compensated by "performance". Sex life marked by control. A general lack of energy and joy; for example, he finds climbing upstairs to the 3rd floor very strenuous. Suggested causes: Control-survival pattern [when our survival is threatened we seek to control reality; it is very difficult to break out again of this pattern, which distorts our focus towards the head and it can persist throughout life], probably at an early stage – embryonically? Mother had murine typhus in late pregnancy – born prematurely? EXERCISE: Write five A4 pages about the listed problems. Find out if they have a common cause. Look at the suppression of your feelings, especially when you are withholding your goodness and friendliness.
>
> Second QOL conversation: He has done his homework: Very intellectual description of something very simple: that he is suffering from anxiety. Anxiety with the following symptoms: 1) his neck is tense, so he cannot sense his feelings; 2) he is shy = social phobia; 3) he is scared of being condemned by others, looked down upon, weighed and found wanting; 4) needs to control feelings and thoughts; 5) needs to keep others at a distance = scepticism, mutual distrust; 6) needs to perform and come out top =

> compensation for fear of social exclusion? On the couch he works on being present in the emotional space, which is much less developed than the physical and mental space. He suffers from anxiety and explains that his mother was never there for him on the emotional level, so /emotional neglect/ from age 3–4 years. Psychosomatic stomach ache as a child. EXERCISE: "Sense your feelings, allowing them space. Sit down for 10 min every day and sense your feelings - egg-timer. Each time that a feeling is provoked in your life you should acknowledge it, allowing it space. Rosen therapy?

Lethargy of this type is not chronic fatigue, but a milder condition. However, this case history is a fine example of how fatigue and lethargy may be due to internal conflicts that can be processed without any major difficulties, if the patient is willing to go along with it.

> **Female, aged 40 years, with chronic fatigue syndrome:** /Essential hypersomnia/ diagnosed in hospital after preclusion of narcolepsy. She has taken antidepressants for a year, stopped two months ago. Things are coming to a head, she says. She has a 12-year-old daughter and has had a boyfriend for seven years. Before him she had another boyfriend for one year, and finally she lived for six years with her child's father, whom she met when she was about 21 years. Depressed during her teens; it was as if she had fallen into a deep, black hole that she could not escape from. She did not know who she was. She wanted to die, but then her mother could not wake up and see her, as the patient longed for her to do. She made herself ill, for instance by walking barefoot through the snow or deliberately falling from a tree so as not to have to go to school. She fantasised about the bus crashing so that she would go to hospital. Sexual abuse by stepfather, who had been in the house since she was two years old. Remembers an episode from when she was about nine, when her stepfather carried her into bed and touched her between the legs. It was a lovely sexual tickle, but she knew she should pretend being asleep. He also tickled her while she was having a bath, but then she laughed and thought that this was how it should be. When she was 16 years old, he watched her secretly in the bath, and one night when she had fallen asleep he undressed her. She dared not refuse him. Talent: To be beautiful. Life purpose: – I am beautiful? I would say /suspected chronic fatigue syndrome/. EXERCISE: Write your life story for 30–60 min daily – what happened? How did you feel? What happened? What decision did you make, if any?) For each situation with a strong emotional content – divorces, assaults, etc. Start from the present. EXERCISE: Accommodate your boyfriend during his mood changes. (Power game? Exercise about this later.) PLAN: To come to the clinic every two weeks; Rosen therapy every two weeks.

## WHIPLASH: WHEN THE BRIDGE TO THE BODY IS BARRED

In our culture, a sprain of the cervical joints may follow a very protracted and sometimes even chronic or life-long course. We now know that a rear-end vehicle collision can cause a number of microscopic injuries to soft tissues and supporting structures in the neck, but it is very strange indeed why neck ligaments and supportive tissues fail to heal properly in some people and why the natural protective immobilization of cervical muscles, serving the purpose of allowing damaged tissues to heal, is never completely healed. There is no apparent reason why those joints should differ from any other joints in the body. Typically, clinical examination of a whiplash patient reveals excessive cervical muscle tension, but no significant damage to ligaments and joints.

A holistic explanation of whiplash injuries comprises two important factors that affect the protective immobilization rendering it permanent following the accident. First, the neck is an especially sensitive region because it is the vulnerable link between head and body and has an important symbolic and emotional meaning. Often, when we shut off our emotions, we experience neck tensions. Second, the muscle tensions storing old, traumatic feelings can shift about in the body. Often during therapy, we experience that one body region has been freed of blockages only to find that another region is equally tense and locked a week later. Tensions migrate from back to neck, from neck to low back, from low back to knees, from knees to feet, effortlessly moving about from one joint to another and from one muscle group to another. When these two circumstances are combined, we obtain a simple psychological explanation of the whiplash syndrome: In order to shut out the painful feelings from his or her personal history, the patient is subconsciously moving old muscle tensions up into the tense muscles in the shoulder and neck. The resulting, very severe, blockage in the neck can have many sequelae such as dizziness, impaired concentration, and depression. That explains why whiplash sometimes causes disabilities that are very difficult to overcome. It is important to understand that the patient often already has emotional problems, which provide reasons for the relocation of muscle tension. Therefore, a patient with chronic whiplash injuries will need a comprehensive regime to sort out his or her personal history with its painful impact on the tensions locking the neck.

In an extremely well-motivated patient, this can sometimes be achieved in one session of consciousness-oriented body therapy, where the patient is made aware of all the problematic aspects. At other times, a long period of therapy is required, perhaps up to 6 months. At any rate, it is treatable if the patient is willing to do the work involved.

> **Male, aged 34 years, with whiplash:** QOL conversation: The patient has suffered from whiplash injury since 1996, accompanied by severe back pain. Migraine since his teens. Has tried NLP, cranio-sacral therapy, healing, a clairvoyant, a chiropractor, etc., but with no lasting effect. Guidance: Basic conflicts of life cause these complaints. PLAN: Rosen method six sessions + gestalt six sessions + reading one book per month – talk to the manager of the QOL Bookstore for advice on literature on personal development, and philosophy of life training. Write autobiography: What happened? How did you feel? Write about all the events in your life when you harboured strong feelings.

In the case above, the treatment went by the book. If the patient does his exercises diligently, he will become better in all likelihood. Below, follow another whiplash story where we describe the patient’s visit instead of presenting the case record. This man in his forties came to our clinic because his wife had read an article about our work in the paper. She thought it was high time he saw someone to get a grip on the condition that had bothered him for the last 5 years and now constituted a threat to the things he valued most in his life — his managerial position heading a department of 25 people, his relationship with his wife after 12 years of marriage, his health and mobility.

He was thick-set, muscular, and strong, but there was something strained and tense about him. He obviously had a sensitive and humane side, but overall gave the impression of being a violent person, one who sought to solve all problems, great and small, with the “hammer he had in his hand”. Three years ago, he went out for a run that turned into a tough experience. He and his partner really exerted themselves, but it was the beginning of a new season and they were not really fit for the effort they made. At home, after the run, he had a terrible headache and went to bed. However, instead of disappearing, his headache increased over the next days, turning into an unbearable pounding in his head, while his neck became increasingly rigid. He had a tingling sensation in the facial skeleton and went to see his physician. Ordinary painkillers provided no relief and he became increasingly tormented. He was admitted to hospital and examined using the finest and most expensive scanners, the state of the art in medical science. However, neurologically, the patient was completely intact with no pathological findings. Nothing could explain his headache.

At the center of his spinal cord, one of the CT scans showed a small stripe that was not supposed to be there. It was suspected that a small vesicle might be pressing on his spinal cord, causing his symptoms. Although now, 1 year after the accident, he was developing a tendency to tingling sensations in his hands, but only when he clenched them or held them in awkward positions. And the vesicle could not explain the headache, the tingling in his brain, or the strange sensations in his face. According to the great medical textbook, that sort of tingling would usually start in one side and not in both sides, as it had in his case.

After all those careful assessments, nobody could explain what was wrong with the patient. We asked him to undress and examined him quite thoroughly. There was nothing of note on the vertebral column, but around the cranial base and superior cervical region, his muscles were very tense. There was a wooden feeling and around his body we found muscles that were extremely hard and dead to the touch. They showed no reaction at all to being touched and were completely tense. Suddenly the “fog lifts”: He has whiplash! Whiplash is a disorder where tension, when induced, can lodge around the neck causing headache and the very symptoms that this patient was suffering from and the lesion need not even be extensive. To relieve the patient of these complaints, we needed to relieve him of the massive tensions in his body. “I believe that you have got whiplash,” I (SV) tell him. “I do not believe that you have a degenerative nervous disorder. Nothing points to that in our opinion. The whiplash injury was caused by all that muscle tension. They tense up to hold on to the pain. I believe that all the tensions we found inside you before are due to the way you try to solve all problems ‘with a hammer’. Instead of having an honest and respectful dialogue with your family and your staff, you try to *hammer* your way through.” “Yes, that is true,” the man says. “They say that when I enter a room everybody shuts up, because they know that either they do as I say, or I give them what for.”

“Couldn’t you consider using a little less power and becoming a slightly more loving person?” I ask him. “Yes, I could consider that. I have thought about it myself,” he says.

“To me, there are two phases in recovering. First, you need to find a way of life so that you do not hurt people. Because that is the pain hidden inside you in all those tense muscles. Then, in order not to become even worse, you will have to find a more loving and respectful way of life, so that you do not hurt other people. You need to open up for your feelings. Neck tension serves to ‘sever’ the head from the body, separating sense and sensibility, so that you can continue your powerful leadership style without feeling how hurtful it is to others. Once you have learnt to behave in a loving and respectful manner and not to hurt people, you will be able to let go of your neck tensions and allow yourself to feel again. Then comes the time when you have to make amends and put everything right again.”

“I hope you do not see me as a wicked person?” he asks. “No, and I do not believe you want to do anything wicked. You just happen to hurt other people. This makes you wicked for all practical purposes and not as good as you might be. What I see is two phases, where first you have to learn a loving and respectful dialogue with the people around you and then clear up all the misery that you have caused.” Surprisingly, the man looks at me as if he understands what I am saying. “I had better go home and practice,” he says.

Perhaps he will be in therapy for years because he cannot let go of his abuse of power towards other people. Perhaps he really understood the essence of it and has decided not to exert his authority over the many people for whom he is responsible. In our opinion, his fate will be decided by his philosophy of life from now on. Faced with the prospect of much-too-early retirement and disaster brewing at home, where his wife is finding him more or less intolerable, he seems to have become highly motivated for understanding and trying to improve. A cosmic setup has been at work. All we need to do is to indicate a way out of the problems and, thus, the road to a new life.

> **Female, aged 48 years, with chronic whiplash:** Car accident 18 months ago with whiplash injury. Since then, the patient can only turn her head through 15 degrees either way and she suffers from headache and impaired concentration. Still working, but it causes her major problems. Time line therapy for the trauma. During her therapy the patient remembers that the other party died, and she feels immense guilt. After thoroughly going over the episode three times she can talk about the trauma. After the session the patient can move her head freely, she laughs and cries and is very happy.

This introduces an important topic of psychosomatics: feelings of guilt. Apparently, the hardest pain in our lives comes from the pain we inflict on others. Killing somebody by accident releases a highly problematic, emotional reaction that is easily repressed, but may have serious consequences for the future well being of that person. In this case, much of the energy behind the patient's whiplash seemed to stem from that feeling of guilt.

## DISCUSSION

Holistic treatment of the highly complex, "new diseases" are often possible with the tools of consciousness-based medicine[78]. The treatment is more complicated and the cure usually takes longer than for less-complex diseases. The problem with these patients is that they have less easily accessible resources than most patients, as they suffer from a combined socio-psycho-physical problem. Often, in our clinical experience, they are depressed and in poor social standing with low confidence and low self-esteem and they have become resigned by not believing that they will get better because they have often already tried most of the specialist and alternative treatments on the market.

To cure them, the most important thing is to coach them to improve their social life by changing their behavior to be of more value to others. Many patients give up when challenged by the physician to improve their quality of life and patients with no initiative or will to get well again are almost impossible to cure, even for the best physician. Holding or care and processing must be especially careful and the contract with the patients must be extremely explicit in order for them to work on their personal development for an hour a day, for 6 or 12 months. Often, they need to read books, write their biography, and many more demanding exercises. If a patient stops doing the prescribed exercises, this must have immediate consequences and the patient must understand that this is not acceptable because if the treatment fails, this is as much a failure for the physician as it is for the patient. This honest and direct approach is often necessary to prevent the patient from taking the easy way out with many of the existential problems that surface during the holistic treatment.

The new diseases can be cured with consciousness-based medicine if the patients are motivated and keep their appointments and all the agreements made. Low responsibility, low personal energy, little joy of life, and limited insight into self and existence are some of the features of the new diseases that make them difficult to cure, also when it comes to holistic medicine. Actually, the patients cannot be cured in the normal sense of the word. They can only cure themselves with coaching by the physician. They must take the initiative and work hard to be well again, they must be their own physician in a way. Due to the fact that they only assume very limited responsibilities or are often "rather emotionally dead", the problem and the trick of treating them is to make a contract the patients are willing and able to keep. Often, treatment of such patients can take years. The important thing is to keep a pace the patient can follow and give the patient a row of small successes and as few failures as possible.

The new diseases are a fantastic challenge for the physician, a unique chance to improve his communication, "holding", and processing skills. Only if the physician refuses to give up on these patients will they have a chance. On the other hand, it is very important that the physician is direct and strong in order not to be a part of the low-responsibility games these patients unconsciously play.

## ACKNOWLEDGMENTS

This study was supported by grants from IMK Almene Fond. The quality of life research was approved by the Copenhagen Scientific Ethical Committee under number (KF)V.100.2123/91.

## BIOSKETCHES

**Søren Ventegodt, MD,** is the director of the Quality of Life Research Center in Copenhagen, Denmark. He is also responsible for a Clinical Research Clinic for Holistic Medicine in Copenhagen and is a popular speaker throughout Scandinavia. He has published numerous scientific or popular articles and a number of books on holistic medicine, quality of life, and quality of working life. His most important scientific contributions are the comprehensive SEQOL questionnaire, the very short QoL5 questionnaire, the integrated QOL theory, the holistic process theory, the life mission theory, and the ongoing Danish Quality of Life Research Survey, 1991–94 in cooperation with the University Hospital of Copenhagen and the late professor of pediatrics, Bengt Zachau-Christiansen, MD, PhD. E-mail: ventegodt@livskvalitet.org. Website: http://www.livskvalitet.org

**Mark Gringols, BPT, MPH,** is a physiotherapist educated at the Department of Physical Therapy, School for Allied Health Professions, Sackler School of Medicine, Tel Aviv University and the Department of Epidemiology, Division of Public Health, Faculty of Health Sciences, Ben Gurion University of the Negev, Beer-Sheva, Israel. Mr. Gringols works as a clinical physiotherapist for the Clalit Health Services in Beer-Sheva and as a clinical instructor at the Recanati School for Community Health Professions, Faculty of Health Sciences, Ben Gurion University of the Negev, Beer-Sheva for students in physiotherapy during their practical clinical period in the community. E-mail: gringols@bezeqint.net

**Joav Merrick, MD, DMSc,** is professor of child health and human development affiliated with the Zusman Child Development Center, Division of Pediatrics and Community Health at the Ben Gurion University, Beer-Sheva, Israel; the medical director of the Division for Mental Retardation, Ministry of Social Affairs, Jerusalem; and founder and director of the National Institute of Child Health and Human Development, Faculty of Health Sciences, Ben Gurion University of the Negev. He has numerous publications in the field of child health and human development, rehabilitation, intellectual disability, disability, health, welfare, abuse, advocacy, quality of life and prevention. Dr. Merrick received the Peter Sabroe Child Award for outstanding work on behalf of Danish Children in 1985 and the International LEGO-Prize ("The Children's Nobel Prize") for an extraordinary contribution towards improvement in child welfare and well being in 1987. E-mail: jmerrick@internet-zahav.net. Website: www.nichd-israel.com
